# Mr. H. Howgrave Graham

**Published:** 1907-05-04

**Authors:** 


### MR. H. HOWGRAVE GRAHAM.

WE expressed our sense of Mr. H. Howgrave Graham's services to the Hospital for Epilepsy and Paralysis in THE HOSPITAL of March 23 last. At the annual meeting on the 27th ultimo, Mr. Charles Drummond, the Treasurer, presented Mr. Graham with a cheque for 400 guineas and a silver salver, as a token of their esteem and as a memento of more than 24 years' faithful service as Secretary to the Hospital, from 1882 to 1907. Mr. Graham, in thanking the governors, exhibited much modesty, but by recalling the facts that he found the hospital in debt and danger, with creditors to pacify, and that he left it with a new hospital built at a cost of £29,000, opened without a penny of debt, and with invested funds exceeding £3,000, proved that the Chairman was justified in describing his services to the hospital as great. Mr. Knapp-Fisher insisted on the indefatigable efforts which Mr. Graham had exhibited, and congratulated the governors that his services would be continued as a member of the committee. He also spoke in grateful recognition of the services frequently rendered by Mrs. Howgrave Graham.

